# Empowering Caregivers: Addressing Needs During the Hospital-to-Home Transition for Sick Children

**DOI:** 10.7759/cureus.74276

**Published:** 2024-11-22

**Authors:** Shramana Ray, Asha P Shetty, Bhagirathi Dwibedi

**Affiliations:** 1 Pediatrics, All India Institute of Medical Sciences, Bhubaneswar, Bhubaneswar, IND

**Keywords:** caregivers, discharge planning, hospital transition, patient education, periodic follow up

## Abstract

Objective: The primary aim of this study is to identify the needs of caregivers who are primarily responsible for caring for their children during the transition from the hospital.

Methodology: A needs assessment survey was conducted among 30 mothers whose children were admitted to the paediatric medicine ward at the All India Institute of Medical Sciences, Bhubaneswar. The researcher utilized a semi-structured questionnaire alongside unstructured discussions to assess the needs and barriers faced by caregivers during hospital transitions.

Results: The findings from the semi-structured questionnaire and informal discussions revealed a range of issues, with the most significant being a lack of knowledge regarding the care domains for children in the post-transition phase. Notably, 100% of caregivers reported a lack of written care instructions as a major barrier to effective care during this period.

Conclusion: The transition period is particularly vulnerable concerning the quality of care for children, and both parents and caregivers encounter numerous challenges when moving from hospital to home. To improve this experience for patients and caregivers, it is essential to focus on caregivers' needs and the barriers they face during the hospital-to-home transition.

## Introduction

Transition refers to any change that occurs as patients move from one area to another, particularly in a hospital setting. In critical care areas, transitions often indicate an improvement in a patient's condition; however, they can also introduce vulnerabilities [1]. Alongside improvement, these transitions carry risks such as ineffective care in the new setting, prolonged hospital stays, and ICU readmissions, which may be associated with increased mortality [2].

Patients are not always ready for transfer due to various factors, including ICU bed shortages, the admission of more critical patients, limited resources, and staff constraints [3]. During these transitions, patients' families often experience significant mental, physical and financial challenges and anxiety as they adapt to a new environment and interact with a different healthcare team. Additionally, families may find themselves needing to take on more caregiving responsibilities at home, while in the ICU, patients typically rely heavily on nursing staff for daily care [4].

Medication management also plays a crucial role during patient transfers. Certain medications administered in the ICU may be discontinued upon transfer, potentially affecting patient stability [5]. Transitions generally occur when patients are clinically recovering from critical illness but still require ongoing acute inpatient care. Inconsistencies in care can be heightened by delayed or night-time discharges.

Patients with chronic illnesses, especially those who have become dependent on new technologies post-discharge, are at greater risk for medical errors, extended hospitalization, and ICU readmissions. The physical shift from a higher-resourced environment to one with fewer resources poses significant challenges for both patients and caregivers [6].

Discharge planning is defined as the systematic preparation of both the patient and their family for a successful transition from the hospital to continued care at home [7]. This process involves identifying the patient’s current and future needs, making informed decisions to address those needs, and coordinating follow-up care. A multidimensional discharge planning approach begins upon the patient’s admission and extends into their home environment. An effective discharge planning program is essential for enhancing the quality of care and ensuring continuity.

The primary aim of this needs assessment survey is to identify the requirements of caregivers who are primarily responsible for caring for their children during transitions. To develop an effective transition model, it is crucial to understand the challenges that parents face during their hospital experience. By gathering insights from parents, we can ensure that our transition model addresses the key areas of need they identify, ultimately smoothing the transition process and providing effective care for their children.

## Materials and methods

This feasibility study utilized a cross-sectional design to evaluate the needs of caregivers preparing for their children’s discharge from the hospital. Conducted over six months, from November 2022 to April 2023, it employed a self-designed needs questionnaire to systematically gather data on the challenges caregivers faced during hospitalization and their requirements for the transition phase.

Participants included primary caregivers of children aged 0 to 12 years who were admitted to the pediatric unit and present during the discharge planning process. Caregivers of children with complex health issues requiring ongoing hospitalization and those unable to communicate effectively in the primary language of the study were excluded.

Data were gathered through structured interviews lasting approximately 15 minutes, conducted prior to discharge. The needs assessment questionnaire comprised both open-ended and closed-ended questions, enabling comprehensive insights into caregivers' experiences and needs. Data collection occurred over a seven-day period in September 2022, involving 30 participants from selected areas of All India Institute of Medical Sciences (AIIMS), Bhubaneswar, India, following verbal consent from all individuals.

The researcher collected demographic data from hospital documentation and established rapport with caregivers to foster open communication regarding their needs. To minimize disruptions to patient care, interviews were scheduled after medical rounds, creating a comfortable environment for caregivers to share their experiences. Confidentiality was maintained throughout the process, with no simultaneous interviews conducted.

Quantitative data from the questionnaires were analyzed using descriptive statistics to summarize caregiver demographics and needs. Chi-square tests were performed to examine associations between caregiver confidence levels and various factors, including age and family support, with a significance threshold set at p < 0.05.

This structured methodology aims to thoroughly evaluate the needs of caregivers during the transition process, providing valuable insights to enhance discharge planning and support services.

## Results

The results of the needs assessment survey provide valuable insights into the demographic and experiential characteristics of the caregivers involved in the study. A total of 30 mothers participated, sharing their experiences and challenges regarding the care of their children during the transition from hospital to home. Key variables assessed to comprehensively understand their needs and barriers included: age, referral source, relationship to the child, prior caregiving experience, previous history of hospitalization or illness in the family, rating of the child’s health, level of caregiver confidence, need for written instructions, and available support at home.

As presented in Table 1, the majority of the children (16, 53.3%) were aged between two and six years, while only one (3.3%) child was aged 7 to 12 years. Among the participants, 17 (43.3%) children were referred to AIIMS from other hospital settings. Notably, of all participants, 30 (100%) were mothers of admitted children. A history of previous hospitalization was reported for 28 (93.3%) children. Regarding family medical history, cardiac issues were noted in 12 (40.0%) families, pulmonary issues in five (16.7%) families, and 13 (43.3%) families reported no history of illness. All mothers rated their children’s health on a scale of 0 to 5, indicating a wide range of health conditions. Of the caregivers, 18 (60%) mothers expressed confidence in their ability to care for their children. Importantly, all mothers indicated a need for written discharge instructions, while only 13 (43.3%) mothers reported having family support available at home.

**Table 1:** Variables in terms of frequency and percentages

Variable	Frequency (%)
Age	
0-6m	7 (23.3)
7-12m	6 (20.00)
2-6y	16 (53.3)
7-12y	1 (3.3)
Total	30 (100)
Referral from hospital	
Yes	17 (43.3)
No	13 (56.7)
Total	30 (100)
Relation with child	
Mother	30 (100)
Previous experience of taking care	
No	18 (60)
Yes	12 (40)
Total	30 (100)
Previous hospitalization	
No	2 (6.7)
Yes	28 (93.3)
Total	30 (100)
History of illness in the family	
No	13 (43.3)
Cardiac	12 (40.0)
Pulmonary	5 (16.7)
Total	30 (100)
Rating of child’s health	
0-5	30 (100)
Level of confidence of caregiver	
No	18 (60)
Yes	12 (40)
Total	30 (100)
Need for written discharge instruction	
Yes	30 (100)
Support available at home	
No	17 (56.7)
Yes	13 (43.3)
Total	30 (100)

**Figure 1:**
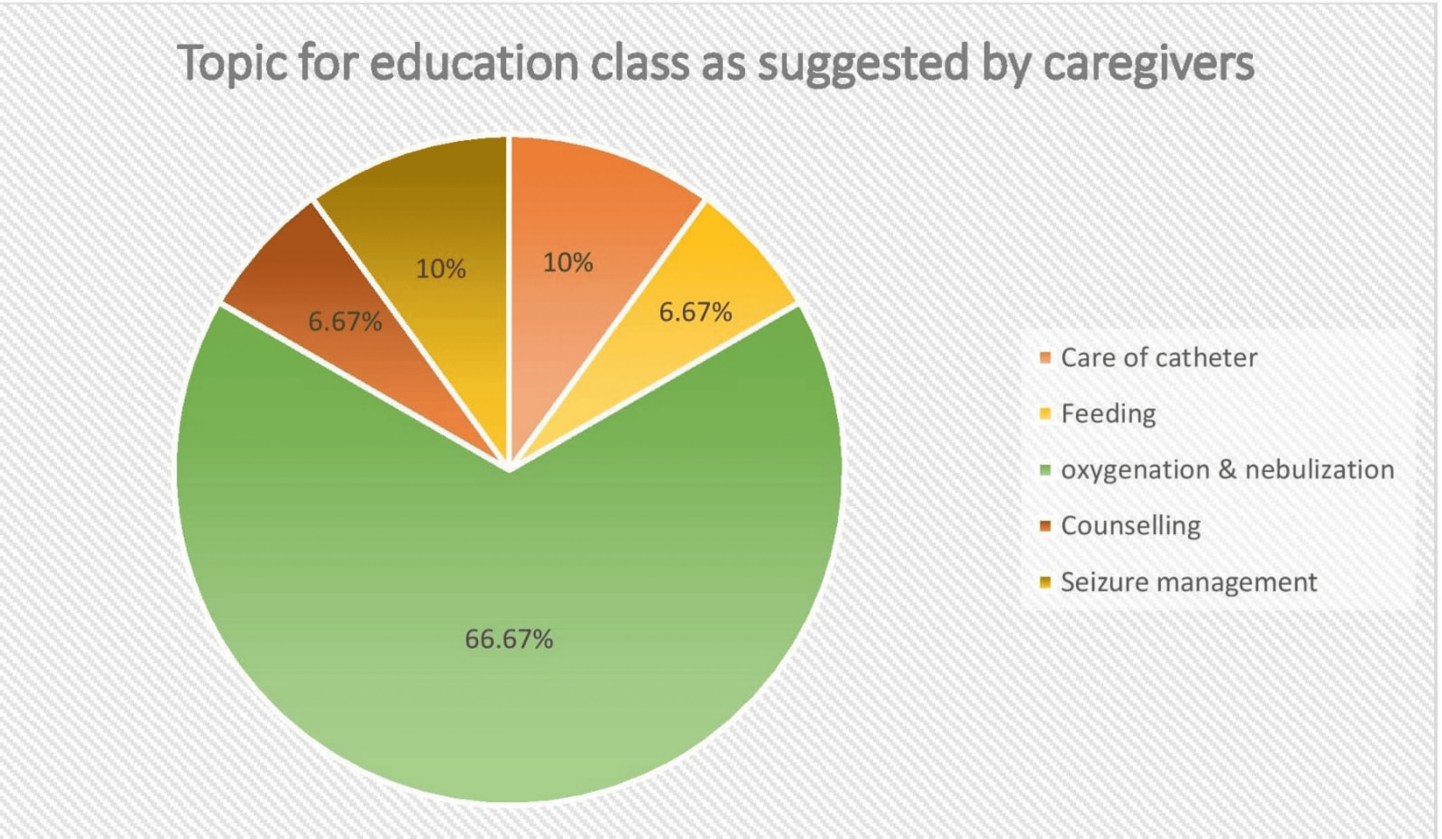
Pie chart showing the percentage of need variables as mentioned by caregivers

Figure 1 illustrates the specific needs articulated by caregivers during the interview process. The pie chart indicates that 20 (66.7%) caregivers emphasized the need for information on “Oxygenation and Nebulization.” Additionally, three (10%) caregivers focused on basic seizure management, while another three (10%) expressed a need for information on “feeding, including nasogastric (NG) tube feeding.” Furthermore, two (6.67%) caregivers requested general counselling on children’s health, and another two (6.67%) caregivers sought clarification on catheter care.

The overall analysis yielded a chi-square value of 6.790 with a p-value of 0.079. This suggests a strong trend indicating that caregiver confidence levels significantly vary across different age groups. Although this p-value is slightly above the conventional threshold for statistical significance (0.05), it highlights a notable association that merits further investigation. Understanding the factors influencing caregiver confidence relative to the child's age is essential for developing effective support strategies (Table 2).

**Table 2:** Associations among age group, family support, and previous hospitalization with caregiver confidence

Variables	Group	Confident (n, %)	Not Confident (n, %)	Total (n, %)	Chi-Square (p-value)
Age Group and Family Support	0-6 Years	5 (71.4)	2 (28.6)	7 (23.3)	χ² = 6.790, p = 0.079
	7-12 Months	1 (16.7)	5 (83.3)	6 (20.0)	
	2-6 Years	11 (68.8)	5 (31.3)	16 (53.3)	
	7-12 Years	0 (0.0)	1 (100.0)	1 (3.3)	
Previous Hospitalization and Confidence	Yes	15 (75.0)	5 (25.0)	20 (66.7)	χ² = 2.738, p = 0.098
	No	2 (20.0)	8 (80.0)	10 (33.3)	

A chi-square value of 2.738 with a p-value of 0.098 was observed, indicating a potential association between previous hospitalization and caregiver confidence. While this finding does not achieve statistical significance, it suggests that caregiver confidence may vary significantly by age group, with those caring for younger children reporting higher levels of confidence.

During the interviews, caregivers shared their concerns and uncertainties about caring for their children at home. One caregiver mentioned, "In the hospital, we are fine because doctors and nurses are there, so if something goes wrong, we can immediately ask for help. But at home, we are not exactly sure how to better take care of the child, so we always have this fear that something might go wrong."

Another caregiver emphasized the lack of hands-on experience during the hospital stay, saying, "We are never fully confident to take care [of the child]. Here, all the caring is done by the nurses - giving medicine, feeding, and other things - but we only observe how they do the procedures. We don’t actually do anything in the hospital. So, when we go back home, sometimes we calculate the medicine wrongly and skip doses."

These qualitative insights highlight the deep-seated concerns caregivers have about their ability to manage the complexities of their child's care once discharged from the hospital. Many expressed a lack of confidence stemming from insufficient hands-on experience during the hospital stay, paired with the overwhelming responsibility of administering medications and performing care procedures alone at home.

In summary, the results of this study indicate that caregiver confidence is influenced by both the age of the child and experiences of previous hospitalization. The quantitative data revealed that caregivers of younger children reported higher confidence levels, while those with previous hospitalization experience also demonstrated increased confidence. The qualitative findings further underscore the need for targeted, practical training and support, as caregivers expressed anxiety about handling their child's care post-discharge, particularly without the direct oversight of healthcare professionals.

These findings emphasize the necessity for tailored support, including comprehensive caregiver education, written discharge instructions, and follow-up care to ensure a smooth transition from hospital to home. This could help mitigate fears and enhance caregiver confidence in managing their child’s health post-discharge.

## Discussion

The transition from hospital to home care presents significant challenges for caregivers, particularly for mothers of children with complex health needs. Our study elucidated a spectrum of concerns that these caregivers face during this critical transition, highlighting the multifaceted nature of their experiences. Participants reported several pressing issues that hinder their ability to provide effective care. For instance, disruptions in electricity supply at home often impede the use of essential medical devices, while the financial strain of ongoing care contributes to heightened stress and feelings of helplessness. Additionally, many mothers expressed confusion regarding the care process, reflecting a lack of understanding that exacerbates their anxiety.

A particularly noteworthy concern identified in our assessment was the absence of written discharge instructions. One caregiver poignantly remarked, “During the transfer process, nurses explain how to perform NG tube feeding and the proper positions for it, but once we are in the ward, there’s no one available to assist. I have to manage feeding my child through this long tube alone, and I constantly worry that I’m not doing it correctly, which could harm my baby.” This statement underscores the critical finding that an inadequate understanding of discharge instructions can leave caregivers feeling ill-equipped and anxious about managing their child's health needs at home.

The emotional toll of caregiving was evident, with many mothers reporting stress, anxiety, and fatigue, often compounded by a lack of family support. This lack of support further amplified their fears of inadvertently causing harm during care. Additionally, caregivers voiced challenges related to recalling procedures and navigating complex technologies, which undermined their confidence in providing care. Addressing these issues is vital to enhance caregiver preparedness and ensure the safety and well-being of the child.

In light of these challenges, mothers articulated specific needs that could significantly enhance their caregiving experience. There was a compelling demand for written care instructions accompanied by images, underscoring the necessity for clear and accessible information for daily management. Counseling sessions were also identified as a potential source of essential emotional support, helping mothers cope with the stress associated with caregiving. Moreover, caregivers expressed a desire for basic information regarding post-discharge routine care and immediate management strategies for emergencies. Such knowledge is crucial for caregivers to feel equipped to handle potential crises, thereby bolstering their confidence in managing their child’s health.

These findings illuminate critical gaps in discharge education for caregivers of pediatric patients, resonating with existing literature on the challenges inherent in effective discharge planning. Ashbrook (2018) emphasizes the importance of clear communication among healthcare providers, noting that while discharge education is viewed as a collective responsibility among nurses, interns, and physicians, the actual communication often remains insufficient. This aligns with our findings, where caregivers expressed a strong need for comprehensive written discharge instructions, suggesting that unclear roles and poor interprofessional communication contribute to caregivers feeling unsupported during transitions [8].

Furthermore, Albrecht et al. (2019) quantified the prevalence of non-comprehension of discharge instructions among older adults, revealing significant non-compliance rates across various domains, including medication management and dietary recommendations. Our study echoes this concern, particularly as caregivers articulated anxiety regarding the management of complex tasks such as NG tube feeding. This indicates that many caregivers may struggle to understand and recall essential post-discharge care instructions, which can adversely affect their child's recovery [9].

Samuels-Kalow et al. (2020) illustrated how language barriers can contribute to dosing errors among non-English-speaking parents, emphasizing the necessity for tailored educational resources that accommodate diverse linguistic backgrounds. Our assessment found that many mothers felt overwhelmed by the complexity of their child’s care, a situation likely exacerbated for those facing language barriers, thus increasing risks in medication administration and other critical tasks [10].

The work of Lee et al. (2021) underscored the demand for post-hospital education among parents of infants and toddlers with congenital heart disease. With 97.1% of parents expressing a desire for educational programs, it is evident that existing discharge processes often fall short of addressing caregivers' educational needs. This sentiment is mirrored in our assessment, where mothers highlighted the urgent need for comprehensive written instructions and follow-up support to facilitate proper care at home [11].

Boulder (2022) implemented an evidence-based practice initiative that exemplifies a proactive approach to enhancing discharge education [12]. By redefining the role of discharge education nurses to include early initiation of instructions and follow-up support, they successfully increased the volume of educational contact without extending patient stays. This model could serve as a valuable framework for our proposed transition care model, emphasizing the necessity of timely, consistent, and clear educational interactions that empower caregivers.

In summary, the collective evidence from our study underscores the urgent need for improved discharge education and support for caregivers. Our findings align with existing literature, indicating that structured communication, tailored educational resources, and a focus on caregiver confidence are essential for facilitating smoother transitions from hospital to home. By addressing these areas, we can enhance the overall quality of care and mitigate the risks associated with post-discharge complications. Since the study was conducted among a specific group of 30 mothers whose children were admitted to the pediatric medicine ward at a single hospital (AIIMS, Bhubaneswar), the findings may not be generalized to all caregivers or children across different regions or healthcare settings.

However, this study is not without limitations. The relatively small sample size may not adequately capture the diverse experiences of all caregivers across different settings, potentially limiting the generalizability of our findings. Additionally, as data were collected from a single institution, the results may not fully reflect the broader context of caregiver experiences in other healthcare environments. Future research should aim to involve a larger and more diverse population to validate these findings and explore the effectiveness of proposed educational interventions.

## Conclusions

The primary objective of this needs assessment was to identify the essential requirements of caregivers whose children are preparing for discharge from the hospital. By utilizing a self-designed needs questionnaire, we effectively pinpointed areas that require greater attention. During the 15-minute interviews, caregivers shared challenges they faced during hospitalization and their needs during their children’s transition phase. A significant emphasis was placed on the necessity for written discharge instructions, as caregivers noted difficulty in recalling procedures after returning home.

Our findings highlight the critical importance of providing comprehensive discharge education to caregivers. The expressed needs for clear, accessible information, including written care instructions and visual aids, underscore the necessity for healthcare providers to enhance communication and support. Additionally, caregivers identified emotional support mechanisms, such as counseling sessions, as vital to managing the stress and anxiety associated with their roles. In conclusion, improving discharge education and support for caregivers is crucial in promoting better health outcomes for children and enhancing the overall caregiving experience. Future efforts should focus on implementing tailored educational interventions that are responsive to the diverse needs of caregivers, ensuring they feel equipped and confident in managing their children’s health post-discharge.

## Figures and Tables

**Figure 2:**
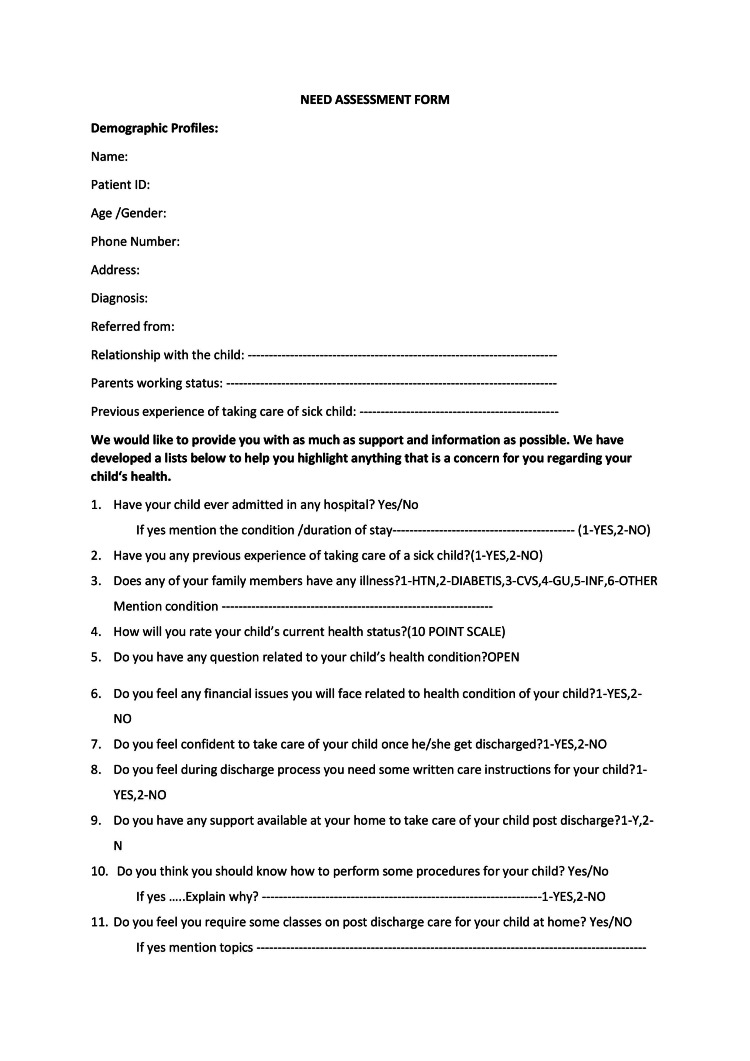
Questionnaire form
